# Genome Sequence of *Bradyrhizobium viridifuturi* Strain SEMIA 690^T^, a Nitrogen-Fixing Symbiont of *Centrosema pubescens*

**DOI:** 10.1128/genomeA.01481-15

**Published:** 2015-12-17

**Authors:** Luisa Caroline Ferraz Helene, Douglas Fabiano Gomes, Jakeline Renata Marçon Delamuta, Renan Augusto Ribeiro, Renata Carolini Souza, Luiz Gonzaga Paula Almeida, Ana Tereza Ribeiro Vasconcelos, Mariangela Hungria

**Affiliations:** a Embrapa Soja, Soil Biotechnology, Londrina, Paraná, Brazil; b UEL, Department of Biochemistry and Biotechnology, Londrina, Paraná, Brazil; c CAPES, SBN, Brasília, Federal District, Brazil; d CNPq, Brasília, Federal District, Brazil; e UFPR, Department of Microbiology, Curitiba, Paraná, Brazil; f LNCC, Labinfo, Petrópolis, Rio de Janeiro, Brazil

## Abstract

SEMIA 690^T^ is a nitrogen-fixing symbiont of *Centrosema pubescens*, and comprises the recently described species *Bradyrhizobium viridifuturi*. Its draft genome indicates that it belongs to the *Bradyrhizobium elkanii* superclade. SEMIA 690^T^ carries two copies of the regulatory *nodD* gene, and the *nod* and *nif* operons resemble those of *Bradyrhizobium diazoefficiens*.

## GENOME ANNOUNCEMENT

A major process for global nitrogen balance results from the symbiotic association of nitrogen-fixing rhizobia and legume plants (1, 2). In the tropics, *Bradyrhizobium* is probably the main symbiotic bacteria (3), and the genus is abundantly found in symbioses with many indigenous legume species (4–6). The genus comprises a variety of species, encompassing stem-nodulating bacteria with photosynthetic and nitrogen-fixing properties (7), to root-nodulating species showing high host-specificity, such as soybean [*Glycine max* (L.) Merrill] (8, 9). Recently, strains SEMIA 690^T^, SEMIA 6387, and SEMIA 6428, symbionts of legumes used for green manure, reforestation, and remediation of degraded areas—and therefore key for green economy—were described as the new species *Bradyrhizobium viridifuturi* (10). Here, we report the draft genome of the type strain of this new species, strain SEMIA 690^T^ (other nomenclature for the strain: CNPSo 991^T^, C 100a^T^, BR 1804^T^, LMG 28866^T^), isolated from *Centrosema pubescens* (Benth.) Kuntze.

To access the bacterial genome sequence, total DNA was extracted using the DNeasy blood and tissue kit (Qiagen) and processed at the Ion PGM platform (Life Technologies) at the LNCC, Petrópolis, Brazil. The FASTQ files were *de novo* assembled by Newbler version 2.9 (Roche). Shotgun sequencing allowed a 28-fold coverage, and the genome analysis revealed that the strain has one circular chromosome. Sequences were submitted to RAST (11), and the genome was estimated at 8,812,829 bp, assembled in 159 contigs. Annotation identified 8,954 coding sequences (CDSs) and 50 tRNAs; 40% of the CDSs were classified in 505 subsystems of the SEED system (11). The major categories were of carbohydrates (14.4%); amino acids and derivatives (13.0); membrane transport (7.7%); cofactors, vitamins, and prosthetic groups (7.7%); and protein metabolism (6.4%).

The draft genome of SEMIA 690^T^ indicates highest similarity (~90%) with *B. elkanii* and *B. pachyrhizi* (10, 12). Among other features, there are 74 CDSs associated with secretion systems, including types I, II, III, and IV. Organization of operons of nodulation (*nod*) and nitrogen fixation (*nif*) genes of SEMIA 690^T^ resemble those of *B. diazoefficiens* USDA 110^T^, but with different levels of similarity. As in USDA 110^T^, there are two copies of the regulatory *nodD* gene (13). We have recently pointed out that the *nodD1* and *nodD2* genes might be relevant, not only for determining host specificity, but also for stress tolerance (14), what could also apply to *B. viridifuturi*, found in tropical soils frequently submitted to environmental stressing conditions. SEMIA 690^T^ also carries *nodZ*, which, as suggested before (15), might play a key role in environmental adaptation, enabling nodulation of a variety of legumes. It remains to be determined if *B. viridifuturi* strains SEMIA 6387 and SEMIA 6428, symbionts of *Acacia* spp. (10), also carry *nodZ*, which is absent in symbionts of *Acacia mearnsii* (15).

**Nucleotide sequence accession numbers**. This whole-genome shotgun project has been deposited at DDBJ/EMBL/GenBank under the following accession numbers: SUBID (SUB1027973), BioProject (PRJNA290320), BioSample (SAMN03890369), and Accession (LGTB00000000). The version described in this paper is the first version.
